# Predictors of Distant Metastasis in Patients with Medullary Thyroid Carcinoma

**DOI:** 10.3390/cancers17193193

**Published:** 2025-09-30

**Authors:** Inmaculada Ros-Madrid, Beatriz Febrero, Rosario Paloma Cano-Mármol, Mercedes Ferrer-Gómez, José M. Rodríguez

**Affiliations:** 1 Endocrinology Service, Virgen de la Arrixaca University Hospital, 30120 Murcia, Spain; inmarosmadrid@gmail.com (I.R.-M.); palomacanomarmol96@gmail.com (R.P.C.-M.); mercedes.ferrer3@carm.es (M.F.-G.); 2 Department of Digestive Surgery, Endocrinology and Organ Transplantation, Biomedical Research Institute of Murcia Pascual Parrilla (IMIB), 30120 Murcia, Spain; 3 Endocrine Surgery Unit, General Surgery Service, Surgery, Virgen de la Arrixaca University Hospital, 30120 Murcia, Spain; jmrodri@um.es; 4 Pediatric, Obstetric and Gynecology Department, Biomedical Research Institute of Murcia Pascual Parrilla (IMIB), University of Murcia, 30120 Murcia, Spain

**Keywords:** medullary thyroid cancer, distant metastasis, prognosis factors

## Abstract

**Simple Summary:**

The presence of distant metastases in patients with MTC determines their survival. However, the clinical behavior of MTC is heterogeneous, which raises the need to identify prognostic factors that allow the risk of metastatic spread to be stratified. The clinical, analytical, and histopathological characterization of patients with MTC is essential for optimizing diagnostic, therapeutic, and follow-up strategies.

**Abstract:**

**Background/Objectives**: The presence of distant metastases is the main cause of death in medullary thyroid carcinoma (MTC). However, due to the rarity of this cancer, few studies have thoroughly analyzed the variables influencing the development of distant metastases. The objective of this study was to evaluate, in patients with MTC, the factors associated with the occurrence of synchronous and metachronous distant metastases. **Methods**: An analytical, observational, retrospective cohort study was conducted at a tertiary hospital. Patients with histologically confirmed MTC, both sporadic and familial (MEN2 syndrome), were included. The influence of epidemiological variables, heredity, complementary tests, surgical factors, histological features, staging, and disease progression was assessed. A univariate comparative analysis was first performed, followed by a multivariate analysis using logistic regression. **Results**: This study included 146 patients, of whom 75% (*n* = 109) had familial MTC. Lymph node involvement at diagnosis was observed in 36% (*n* = 52). During follow-up, distant metastases developed in 14% (*n* = 21) of patients, including five cases present at the time of diagnosis. The median follow-up was 214 months (IQR 106–289). The presence of distant metastases was associated with an increased risk of mortality. Factors associated with distant metastases included age, calcitonin level, hereditary status, lymph node involvement, and overall stage. In multivariate analysis, the lymph node ratio (LNR) remained the only significant predictor (OR 29.124). **Conclusions**: Several variables were related to the presence of distant metastases. Among them, the LNR emerged as the independent predictor of both synchronous and metachronous distant metastases.

## 1. Introduction

Patients with medullary thyroid carcinoma (MTC) present distant metastases in 6.6–23% of cases [1,2,3,4,5,6,7], most frequently located in the bone (40–56%), liver (45–83%), lung (25–62%), and brain (4%) [2,3,8]. Around 10% of patients with a palpable thyroid nodule already have metastatic disease at diagnosis [9,10]. After potentially curative surgery, 1.17–15% may develop metastatic recurrence [11,12,13]. While structural cervical recurrence is typically diagnosed within the first year, the mean time to the development of distant metastases is longer, occurring at around three years [1].

The presence of distant metastases is the leading cause of death in MTC [6]. Patients with distant metastases have a 3.99–12.3-fold higher risk of disease-specific mortality [2,9,10,11,14,15,16,17,18], and a 2.3–8.14-fold higher risk of overall mortality over time [10,13,14,19,20,21,22,23]. Disease-specific survival in MTC (DSS-MTC) at 3 and 5 years is 61.6% and 51.9%, respectively, in patients with distant metastases, compared with 96.3% and 94.7% in those without metastases [4]. However, given the rarity of this cancer, few studies have analyzed in detail the variables associated with the development of distant metastases [4,8,24,25,26,27]. To date, some studies, such as that of Lee et al. [24], have explored this aspect in depth, with the latter developing a nomogram to stratify patients into low-, intermediate-, or high-risk groups, which correlates with overall survival (OS). In this context, the aim of the present study was to determine the variables influencing the occurrence of synchronous and metachronous distant metastases in patients with MTC.

## 2. Materials and Methods

This was an analytical, observational, retrospective cohort study. We included consecutive cases of histologically confirmed sporadic or hereditary MTC attended at the Outpatient Clinics of Endocrinology and Endocrine Surgery of the Virgen de la Arrixaca University Hospital (Murcia, Spain) between 2022 and 2024. Although patient recruitment was carried out during this period, clinical data were collected retrospectively from the time of diagnosis, covering cases diagnosed from 1979 to 2023.

### 2.1. Variables Analyzed

- Epidemiological: sex and age.
- Hereditary: sporadic/familial.

For familial cases:

▪ Phenotype (MCT with/without hyperparathyroidism (HP), pheochromocytoma or paraganglioma).
▪ Mutation (exon, genotype, affected protein).
▪ Mutation risk level according to ATA 2009 [28].

- Complementary examinations:

(1) Laboratory:

- Calcitonin (Ct, pg/mL).

Postoperative Ct determination: until 2003, Ct was measured by immunoradiometric assay (CT-US IRMA, DiaSource, Louvain-la Neuve, Belgium). Normal basal Ct values were defined as <10 pg/mL. Since 2003, Ct has been determined by chemiluminescence (immunoluminometric assay) with Elecsys^®^ hCT immunoassay (Roche Diagnostics, Mannheim, Germany). Normal reference values: <9.82 pg/mL for women, <14.3 pg/mL for men.

For data analysis, the results were standardized using the correlations reported by Schiettecatte et al. [29], where regression analysis (Passing-Bablok) showed good correlation for concentrations > 30 pg/mL, while for <30 pg/mL, the following conversion was required:

Ct [Elecsys] = 1.03 × Ct [IRMA] − 0.88.

- CEA (ng/mL).

Determined by chemiluminescence (Cobas e601–e602, Roche Diagnostics, Mannheim, Germany). Normal values: <4.7 ng/mL.

(2) Imaging studies

- Thyroid ultrasound.

◦ Findings (unilateral nodule/unilateral nodules/bilateral nodules/normal/not available).
◦ Nodule size
◦ Suspicious lymphadenopathy (yes/no).

- Surgery-related variables

(1) Age at surgery.
(2) Suspicious lymphadenopathy (yes/no).
  - Prophylactic surgery: In asymptomatic patients carrying the RET mutation with no CMT according to clinical and radiological criteria (no nodules or nodules < 5 mm identified by ultrasound; and no suspicious lymphadenopathy) [30,31], prophylactic surgery was considered if performed in the first year of life in the case of MEN2B, before the age of 5 in the case of high-risk MEN2A.
  - Early surgery: This is surgery performed on asymptomatic RET mutation carriers with no CMT according to clinical and radiological criteria (no nodules or nodules < 5 mm identified by ultrasound; and no suspicious lymphadenopathy) [30,31]. This surgery is performed after the first year of life in MEN 2b syndrome and after 5 years of age in high-risk MEN2A syndrome.
  - Curative surgery: this occurs when the disease has been identified, and the aim of the intervention is to achieve a cure.
  - Palliative surgery: performed when the disease is not curable, and the aim is to relieve symptoms.

(3) Surgical technique (total thyroidectomy with/without central lymph node dissection, with/without unilateral/bilateral lateral lymph node dissection), according to ATA recommendations.
(4) Complications (yes/no).
  - Postoperative hypoparathyroidism: inappropriate PTH production.
    ◦ Transient: resolved within 6 months.
    ◦ Permanent: persistent beyond 6 months.
  - Recurrent laryngeal nerve paralysis (transitory/permanent).
  - Others: hematoma, infection, chylous fistula, jugular vein thrombosis, hemidiaphragm paralysis, respiratory failure.

- Histological variables

(1) Tumor size (mm).
(2) Focality (unifocal/multifocal).
(3) Capsular invasion.
(4) Vascular invasion.
(5) Lymphatic invasion.
(6) Tumor necrosis.
(7) Number of mitoses per 10 high power field (HPF).
(8) Ki67.
(9) High-risk histology: ≥1 of the following: tumor necrosis, >5 mitoses/2 mm^2^, Ki67 > 5% [32].
(10) Desmoplasia: fibrotic tissue surrounding tumor tissue, absent in normal thyroid parenchyma [33].

- Staging

(1) Nodal involvement (yes/no).
(2) Central node involvement.
(3) Lateral nodal involvement.
(4) Ipsilateral lateral nodal involvement.
(5) Contralateral lateral nodal involvement.
(6) Number of pathological nodes (0/1/>1).
(7) Number of pathological lymph nodes.
(8) Number of lymph nodes removed.
(9) LNR (lymph node ratio): proportion of metastatic lymph nodes to the total number of lymph nodes removed.
(10) TNM stage (AJCC, 8º Edition) [34].
(11) Stage (I/II/III/IVa/IVb/IVc).

- Evolution

(1) Ct at 6–12 months (pg/mL).
(2) BFS (biochemical-free survival) (months): time from initial surgery to biochemical recurrence.
(3) OS (overall survival, months).
(4) Death and cause.

### 2.2. Statistical Analysis

Statistical analysis was performed with IBM SPSS Statistics v.28.0 for MAC (SPSS Inc., Chicago, IL, USA). Survival was analyzed using Kaplan–Meier curves, and the log-rank test was used to assess the impact of distant metastases on OS. Comparative univariate analysis was performed using the Chi-squared test, Mann–Whitney U test, and Kruskal–Wallis test. Multivariate logistic regression was then applied to identify independent predictors of distant metastases in the entire sample and in the familial CMT subgroup. The association between preoperative Ct and distant metastases was assessed using logistic regression with restricted cubic splines (four knots at default quantiles, rms package in R), allowing for non-linear effects.

## 3. Results

Among the 146 patients initially included, 51% (*n* = 74) were women, with a mean age of 34.51 years. In total, 25% (*n* = 37) had sporadic MTC, while 75% (*n* = 109) had hereditary MTC (106 MEN2A, 3 MEN2B). Lymph node involvement at diagnosis was observed in 36% (*n* = 52), of which 46% (*n* = 45) were located in the central compartment. Among the 65 patients who underwent lateral lymphadenectomy, ipsilateral involvement was present in 48% (*n* = 36) and contralateral involvement in 28% (*n* = 18). Preoperatively, the median Ct was 242 pg/mL.

A total of 14% (*n* = 21) of patients had distant metastases, of which five had metastases already present at diagnosis. All patients underwent surgery: 33% (*n* = 48) with total thyroidectomy (TT), 23% (*n* = 33) TT with central lymph node dissection (CLND), 9% (*n* = 13) TT with CLND and unilateral neck lateral dissection (ULLND), and 35% (*n* = 52) TT with CLND and bilateral neck lateral dissection (BLLND). Regarding systemic or adjuvant therapies, 62% (*n* = 13) received somatostatin analogues, 38% (*n* = 8) tyrosine kinase inhibitors, 38% (*n* = 8) radiotherapy, 14% (*n* = 3) chemotherapy, and 5% (*n* = 1) hepatic chemoembolization.

**Figure 1:**
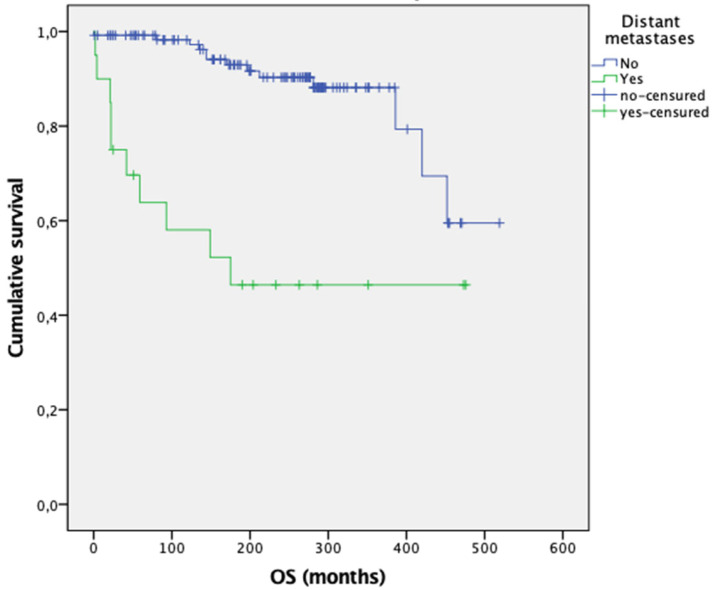
Overall survival according to the presence of distant metastases at the end of follow-up. Log Rank: x^2^ = 22.222 (*p* < 0.001). N = 144 (deaths = 23). Median OS (months): 449 for patients without distant metastases vs. 254 for patients with distant metastases.

The median follow-up was 214 months (IQR 106–289). During this period, 16% of patients (*n* = 23) died (Supplementary Table S1). Patients who developed distant metastases had a significantly higher risk of death, with a mean OS of 254 months, compared to 449 months in those without distant metastases (*p* < 0.001) (Figure 1).

### 3.1. Univariate Analysis

When comparing epidemiological and preoperative clinical characteristics, as well as complementary tests, between patients with and without distant metastases (Table 1), those who developed distant metastases were older (44 vs. 33 years; *p* = 0.006), had higher preoperative Ct levels (median 1500 pg/mL [IQR 706–6657] vs. 116 pg/mL [IQR 48–664]; *p* < 0.001) and higher CEA levels (51.5 vs. 5.8 ng/mL; *p* = 0.005), and more frequent lymph node involvement on ultrasound (52% vs. 14%; *p* < 0.001). The range of Ct levels in patients with distant metastases was 70–53,700 pg/mL, compared with 0.77–14,860 pg/mL in patients without metastases.

In addition, patients with sporadic MTC had a higher proportion of distant metastases than those with familial MTC (32% vs. 8%; *p* = 0.001) (Table 1). Among patients with familial MTC, no differences in the occurrence of distal metastases were observed according to phenotype or specific genetic mutation (Table 2). No statistically significant association was found in terms of treatment and complications (Table 3).

With regard to staging (Table 4), patients who developed distant metastases had a higher proportion of advanced disease: T3–4 tumors (35% vs. 8%; *p* < 0.001), N1b nodal stage (58% vs. 21%; *p* = 0.004), overall lymph node involvement (67% vs. 30%, *p* = 0.002), and stage III-IV disease (76% vs. 30%; *p* < 0.001). They also presented more pathological lymph nodes (7.5 vs. 0, *p* < 0.001) and a higher LNR (0.62 vs. 0.04, *p* < 0.001). No other variables analyzed reached statistical significance (*p* > 0.05).

In relation to the anatomopathological characteristics (Table 5), patients with distant metastases had significantly larger tumors (median 17 vs. 9 mm; *p* = 0.001). The rest of the variables analyzed were not statistically significant (*p* > 0.05).

With regard to the evolution of patients with distant metastases (Table 6), a higher proportion of deaths was observed (50% vs. 10%, *p* < 0.001), which was related to neoplastic progression of MTC (90% vs. 15%, *p* = 0.001).

### 3.2. Multivariate Analysis of Predictors of Distant Metastases in Patients with MTC

**Figure 2:**
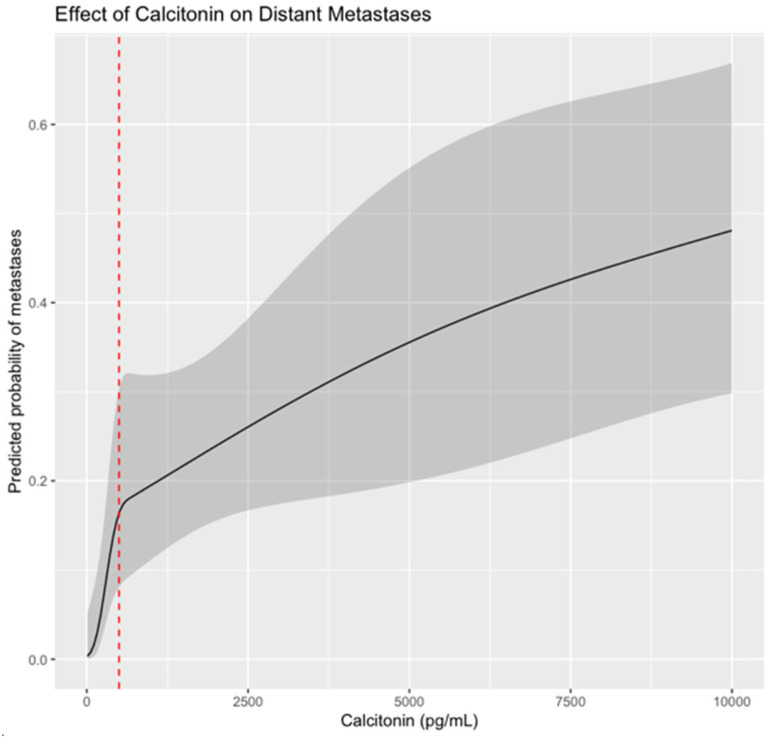
Predicted probability of distant metastases according to preoperative serum Ct, modeled using restricted cubic splines in a logistic regression framework. The red dashed line indicates 500 pg/mL, a clinically relevant threshold. Shaded areas represent 95% confidence intervals.

The association between preoperative serum calcitonin and distant metastases was assessed using logistic regression with restricted cubic splines. The predicted probability of distant metastases increased progressively with higher Ct levels. As shown in Figure 2, the risk rises sharply around the clinically relevant threshold of 500 pg/mL (marked by a red dashed line). Across the observed range of Ct (28–1701 pg/mL), the odds ratio between the highest and lowest values was 62.8 (95% CI: 4.35–906.7), reflecting a strong association with distant metastases.

In the multivariate analysis, the following variables were included: age at diagnosis, heredity, preoperative Ct ≧ 500 pg/mL, tumor size, T stage, N stage, lymph node involvement, ipsilateral lateral lymph node involvement, contralateral lymph node involvement, LNR, and overall tumor stage. Variables that were significant in the univariate analysis but not included in the model due to a high proportion of missing data were CEA, vascular invasion, lymphatic invasion, mitotic count, and Ki67. The only variable that remained statistically significant in the multivariate model was LNR, which was associated with a 16.460 fold higher risk of developing distant metastases (Table 7 and Supplementary File, Table S2).

A logistic regression analysis was performed in the subgroup of patients with familial MTC, including age at diagnosis, genetic mutation, preoperative calcitonin level, tumor size, T stage, N stage, lymph node involvement (ipsilateral, contralateral, and overall), lymph node ratio (LNR), overall tumor stage, and mutation risk level according to the 2009 ATA classification. Among these variables, only Ct ≧ 500 pg/mL remained significantly associated with the presence of distant metastases, while the mutation risk level and the other factors did not persist as significant predictors (Table 8).

## 4. Discussion

The results of this study show that 14% of patients with MTC developed distant metastases, a proportion consistent with previous reports in the literature (6.6–23%) [1,2,3,4,5,6,7]. The identification of variables that increase the likelihood of distant metastases is of particular interest, as this condition significantly worsens OS by increasing the risk of death due to MTC progression, as observed both in this study (see Figure 1) and by others [10,13,14,20,21,22,23].

The presence of distant metastases has been associated with older age at diagnosis, consistent with previous reports [4,24,26]. However, in the present study, age did not emerge as an independent risk factor, unlike in the study by Lee et al. [24], where each incremental year of age was associated with a 1.57-fold higher risk of metastatic involvement. Notably, the role of age has been assessed heterogeneously in the literature. For instance, Chen et al. [4] evaluated the impact of age using a cutoff of >55 years, reporting an Odds Ratio (OR) of 3.226. In contrast, Su et al. [26] analyzed age in three categories (≤18 years, 19–55 years, and >55 years), showing a significantly higher risk at the extremes (<18 years: OR 4.98; >55 years: OR 3.203) compared to the intermediate group. Conversely, in the study by Pazaitou et al. [25], age was not an influential factor in the presence of distant metastases. Overall, there is no clear consensus regarding the role of age in the development of distant metastases in MTC.

Heritability, specifically the presence of sporadic MTC, was initially associated with a poorer prognosis due to a higher risk of distant metastases; however, this was not confirmed in the multivariate analysis. This initial association is likely attributable to the fact that patients with sporadic MTC are usually diagnosed at an older age compared with those detected through familial screening. In the published studies analyzing factors related to metastatic spread, the influence of heritability was not specifically addressed. Nevertheless, multifocality—more frequently observed in familial MTC—was considered, although no significant association was found between multifocality and metastatic involvement [4,24,25]. This study included a high proportion of patients with familial MTC (75%), which enabled a logistic regression analysis within this subgroup to explore potential associated factors. In this analysis, only calcitonin ≧ 500 pg/mL remained a significant variable. Importantly, 75% of these patients harbored the exon 11 p.C634T mutation, meaning that the findings are mainly representative of this specific subgroup. Since this mutation is commonly associated with a more aggressive clinical course, our results provide relevant insights for risk stratification and therapeutic decision-making in this particular context.

Patients with distant metastases exhibited elevated basal Ct levels (median 1500 pg/mL). In fact, ESMO recommends assessing for distant disease in patients with Ct levels above 500 pg/mL, based on the study by Machens et al. [35], which reported no cases of distant disease with levels below this threshold. In our cohort, 24% of patients with distant metastases already presented with them at diagnosis. Notably, despite this, we observed cases in which patients reached Ct levels above 500 pg/mL—even up to 14,000 pg/mL—without evidence of metastases, although all patients who eventually developed metastases had Ct levels > 500 pg/mL. The spline analysis made in our study highlights that the risk does not increase linearly and underscores the importance of considering non-linear effects, particularly around decision thresholds such as 500 pg/mL. Because of this, we considered this variable as dichotomous in the multivariate logistic regression analysis, observing how it remained significant with an OR of 7.985. These findings support the concept that Ct serves more as a marker of tumor burden rather than disease extent.

The median CEA level in patients with metastatic involvement was 51.5 ng/mL; however, other studies [35] have proposed a cutoff value of 100 ng/mL, which is associated with a >75% risk of distant metastases, suggesting that this threshold may need to be reconsidered. Although CEA represents a potential predictor of distant metastasis, it was not included in the multivariate analysis due to missing data in a large proportion of patients.

Tumor size, whether analyzed as a continuous variable or by T stage, was associated with the occurrence of distant metastases. However, it did not remain an independent variable, consistent with the findings of Pazaitou et al. [25]. This contrasts with the results reported by Lee et al. [24], who observed that the risk of metastatic involvement increased 1.8-fold for each unit increase in tumor size. Furthermore, other studies [4,8,26] have demonstrated a significant association between T stage and the development of distant metastases, with progressively higher risks: T2 (OR 3.9), T3 (OR 5.2–8.3), T4 (OR 8.58), or combined T3–4 stage (OR 3.72). In the studies by Lee et al. [24] and Machens et al. [8], the mean tumor sizes were 2 cm and 1.8 cm, respectively, whereas in Chen et al. [4], more than 50% of patients presented with tumors >2 cm. In our cohort, the median tumor size was 1 cm, which may have limited the ability to demonstrate an independent association between tumor size and the risk of distant metastasis, as this risk tends to increase predominantly in larger tumors.

Lymph node involvement significantly increases the risk of distant metastases, as observed in this and other studies [24,26] (OR 4.25–5.52). However, another study [4] evaluated lymph node involvement using both the N stage and the lymph node ratio (LNR). The LNR has been proposed to assess nodal involvement with less influenced from the extent of surgery [4]. In addition, it could predict DFS [36,37], PFS [38], OS [37,39]. This factor has been independently associated with the development of distant metastases [1,4]. Similarly, in our study, the LNR was the only variable independently associated with the risk of distant metastases (OR 29.12). In Chen et al. [4], patients with an LNR >0.4 showed an OR of 3.08. Prinzi et al. [1] suggested that an LNR >0.12 was a reliable cutoff to predict the risk of distant metastases, since these patients exhibited a 34% risk compared to 0% in those with an LNR <0.12.

Regarding mitotic count, Ki67, and tumor necrosis, no significant differences were observed between patients with or without metastases. In contrast, Xu et al. [27] reported that patients with necrosis, high Ki67 expression, and a higher mitotic count exhibited reduced metastasis-free survival; in fact, risk classification based on these features was one of the variables independently predicting metastasis-free survival. In our study, these variables could not be adequately assessed due to the small sample size and missing data.

With respect to desmoplasia, although no significant differences were identified, it is noteworthy that none of the patients without desmoplasia developed metastatic disease. Similarly, this observation has been reported in other studies [40,41]. Therefore, further investigation of this feature in patients with MTC is warranted.

Lymphatic and vascular invasion are two variables that have been associated with metastatic involvement. However, in our analysis, this relationship could not be confirmed, as it was assessed in only a limited proportion of the sample. Future studies should address these variables in a more systematic manner to obtain more conclusive results. One of the main limitations of this study is its retrospective design and the heterogeneity of the included cohort. Our study included 75% of patients with familial MTC, whereas previous studies typically include a much smaller proportion. Therefore, our findings provide valuable information specifically related to this patient group, in contrast to other studies where familial cases represent only around 30% of the cohort [13,25]. Nevertheless, 140 patients were analyzed, a considerably larger sample compared to other previously published studies. Further confirmation of these findings through multicenter studies with larger sample sizes is warranted, along with continued investigation into the pathophysiological mechanisms underlying the biological behavior of MTC, with the aim of improving prognostic stratification and personalizing therapeutic management. Based on the literature and the results of this study, LNR emerges as a potential tool that could help guide treatment strategies, representing a relevant area for future research.

## 5. Conclusions

Several variables are associated with the presence of distant metastasis in patients with MTC. Among these, the most relevant and independently predictive factor is the LNR, which is strongly associated with the development of both synchronous and metachronous distant metastases.

## Figures and Tables

**Table 1:** Preoperative clinical, epidemiological, and laboratory characteristics according to the presence of distant metastases.

Patients with MTC		Metastases 14% (*n* = 21)	No Metastases 86% (*n* = 125)	*p*
Sex ^1^	Woman (51%, *n* = 75)	10 (48%)	65 (52%)	0.710
	Man (49%, *n* = 71)	11 (52%)	60 (48%)	
Age at diagnosis ^2^	Ages (median)	44 (*n* = 21)	33 (*n* = 125)	**0.006**
Heritability ^1^	Sporadic (25%, *n* = 37)	12 (32%)	25 (68%)	**0.001**
	Familial (75%, *n* = 109)	9 (8%)	100 (92%)	
Ct ^2^	pg/mL (median)	1500 (*n* = 17)	116 (*n* = 119)	**<0.001**
CEA ^2^	ng/mL (median)	51.5 (*n* = 9)	5.8 (*n* = 79)	**0.005**
Thyroid ultrasound ^1^	Unilateral nodule (42%, *n* = 52)	12 (63%)	40 (38%)	0.090
	Bilateral nodules (30%, *n* = 37)	5 (26%)	32 (31%)	
	Normal (28%, *n* = 35)	2 (11%)	33 (31%)	
	Suspicious adenopathy (19%, *n* = 28)	11 (52%)	17 (14%)	**<0.001**
	No (66%, *n* = 96)	8 (38%)	88 (70%)	
	Unknown (15%, *n* = 22)	2 (10%)	20 (16%)	
Thyroid nodule size in the ultrasound ^1^	Millimeters (median)	17 (*n* = 15)	15 (*n* = 66)	0.398

Values are presented as frequences with percentages or medians. Statistical differences were assessed using Chi-squared test ^1^ (sex, heritability, thyroid ultrasound, thyroid nodule size in the ultrasound), Mann–Whitney test ^2^ (age, Ct, CEA). Values in bold represent significance. Ct: calcitonin; CEA: carcinoembryonic antigen.

**Table 2:** Clinical and genetic characteristics of patients with familial MTC according to the presence of distant metastases.

Patients with Familial MTC		Metastases (*n* = 9)	No Metastases (*n* = 100)	*p*
Phenotype—Pheochromocytoma/ paraganglioma ^1^	Yes (58%, *n* = 63)	4 (44%)	59 (59%)	0.489
	No (42%, *n* = 46)	5 (56%)	41 (41%)	
Phenotype—HP ^1^	Yes (12%, *n* = 13)	0 (0%)	13 (13%)	0.596
	No (88%, *n* = 96)	9 (100%)	87 (87%)	
Genetic mutation ^1^	Very high risk (3%, n = 3)	0 (0)	3 (3%)	-
	High risk (92%, *n* = 98)	5 (71%)	93 (93%)	
	Moderate risk (5%, *n* = 6)	2 (29%)	4 (4%)	

Values are presented as frequences with percentages. Statistical differences were assessed using Chi-squared test ^1^ (phenotype-pheochromocytoma/paraganglioma, phenotype-HP, genetic mutation). HP: primary hyperparathyroidism. “-“ The *p*-value was not calculated due to insufficient numbers in some categories.

**Table 3:** Differences in treatment between patients based on the appearance of distant metastases.

Patients with MTC		Metastases 14% (*n* = 21)	No Metastases 86% (*n* = 125)	*p*
Treatment ^1^	Early/prophylactic (25%, *n* = 36)	0 (0%)	36 (29%)	-
	Curative (72%, *n* = 105)	16 (76%)	89 (71%)	
	Palliative (3%, *n* = 5)	5 (24%)	0 (0)	
Surgical technique ^1^	Total thyroidectomy (TT) (33%, *n* = 48)	4 (19%)	44 (35%)	-
	TT + CLND (22%, *n* = 33)	3 (14%)	30 (24%)	
	TT + CLND + ULLND (8%, *n* = 12)	5 (24%)	7 (6%)	
	TT + CLND + BLLND (36%, *n* = 52)	8 (38%)	44 (35%)	
	Somatostatin analogues (1%, *n* = 1)	1 (5%)	0 (0)	
Complications ^1^	Yes (33%, *n* = 48)	9 (43%)	39 (31%)	0.293
	No (67%, *n* = 98)	12 (57%)	86 (69%)	
Hypoparathyroidism ^1^	Transitory (21%, *n* = 30)	7 (35%)	23 (18%)	0.187
	Permanent (8%, *n* = 13)	1 (5%)	10 (10%)	
	No (71%, *n* = 103)	12 (60%)	91 (72%)	
Recurrent laryngeal nerve paralysis ^1^	Transient (3.5%, *n* = 5)	2 (10%)	3 (2%)	0.205
	Permanent (3.5%, *n* = 5)	1 (5%)	4 (3%)	
	No (93%, *n* = 134)	17 (85%)	117 (95%)	

Values are presented as frequences with percentages. Statistical differences were assessed using Chi-squared test ^1^ (treatment, surgical technique, complications, hypoparathyroidism, recurrent laryngeal nerve paralysis). TT: total thyroidectomy. CLND: central lymph node dissection, ULLND: unilateral neck lateral dissection, BLLND: bilateral neck lateral dissection. “-” The *p*-value was not calculated due to insufficient numbers in some categories.

**Table 4:** Differences in staging between patients based on the appearance of distant metastases.

Patients with MTC		Metastases 14% (*n* = 21)	No Metastases 86% (*n* = 125)	*p*
T ^1^	T1–2 (88%, *n* = 128)	13 (65%)	115 (92%)	**<0.001**
	T3–4 (12%, *n* = 17)	7 (35%)	10 (8%)	
N ^1^	N0 (28%, *n* = 41)	2 (9%)	39 (31%)	**0.004**
	N1a (9%, *n* = 13)	2 (9%)	11 (9%)	
	N1b (26%, *n* = 38)	12 (58%)	26 (21%)	
	Nx (37%, *n* = 54)	5 (24%)	49 (39%)	
Nodal involvement ^1^	Yes (35%, *n* = 51)	14 (67%)	37 (30%)	**0.002**
	No/no lymphadenectomy (65%, *n* = 95)	7 (33%)	88 (70%)	
Central nodal involvement ^1^	Yes (15%, *n* = 15)	2 (12%)	13 (16%)	1
	No (85%, *n* = 82)	14 (88%)	68 (84%)	
Ipsilateral lateral nodal involvement ^1^	Yes (36%, *n* = 35)	12 (75%)	23 (28%)	**<0.001**
	No (64%, *n* = 63)	4 (25%)	59 (72%)	
Contralateral lateral nodal involvement ^1^	Yes (35%, *n* = 18)	6 (75%)	12 (27%)	**0.015**
	No (65%, *n* = 34)	2 (25%)	32 (73%)	
Stage ^1^	Stage I and II (63%, *n* = 92)	5 (24%)	87 (70%)	**<0.001**
	Stage III and IV (37%, *n* = 54)	16 (76%)	38 (30%)	
Number of lymph nodes removed ^2^	(median)	13.5 (*n* = 14)	18 (*n* = 68)	0.739
Number of pathological lymph nodes ^2^	(median)	7.5 (*n* = 16)	0 (*n* = 81)	**<0.001**
LNR ^2^	(median)	0.62 (*n* = 14)	0.04 (*n* = 68)	**<0.001**

Values are presented as frequences with percentages or medians. Statistical differences were assessed using Chi-squared test ^1^ (T, N, nodal involvement, central nodal involvement, ipsilateral lateral nodal involvement, stage), and Mann–Whitney test ^2^ (number of lymph nodes removed, number of pathological lymph nodes, LNR). Values in bold represent significance. LNR: lymph node ratio. T: Tumor. N: nodes.

**Table 5:** Anatomopathological characteristics of patients according to the presence of distant metastases.

Patients with MTC		Metastases 14% (*n* = 21)	No Metastases 86% (*n* = 125)	*p*
Size ^2^	Millimeters (median)	17 (*n* = 20)	9 (125)	**0.001**
Focality ^1^	Unifocal (31%, *n* = 46)	8 (39%)	38 (30%)	0.402
	Multifocal (66%, *n* = 95)	12 (57%)	83 (67%)	
	Unknown (3%, *n* = 5)	1 (4%)	4 (3%)	
Capsular invasion ^1^	Yes (8%, *n* = 12)	7 (33%)	5 (4%)	**-**
	No (7%, *n* = 10)	1 (5%)	9 (7%)	
	Unknown (85%, *n* = 124)	13 (62%)	111 (89%)	
Vascular invasion ^1^	Yes (13%, *n* = 19)	7 (33%)	12 (9%)	**-**
	No (32%, *n* = 47)	1 (5%)	46 (37%)	
	Unknown (55%, *n* = 80)	13 (62%)	67 (54%)	
Lymphatic invasion ^1^	Yes (16%, *n* = 24)	6 (29%)	18 (14%)	**-**
	No (28%, *n* = 41)	0 (0)	41 (33%)	
	Unknown (56%, *n* = 81)	15 (71%)	66 (53%)	
Tumor necrosis ^1^	Yes (1%, *n* = 1)	1(5%)	0	**-**
	No (31%, *n* = 46)	7 (33%)	39 (27%)	
	Unknown (68%, *n* = 99)	13 (62%)	86 (69%)	
Number of mitoses ^2^	(median)	1 (*n* = 8)	0 (*n* = 33)	0.035
Ki67 ^2^	% (median)	1 (*n* = 8)	1 (*n* = 33)	0.330
Desmoplasia ^1^	Yes (24%, *n* = 35)	9 (43%)	31 (25%)	-
	No (6%, *n* = 9)	0 (0)	17 (14%)	
	Unknown (70%, *n* = 102)	12 (57%)	77 (61%)	
High risk ^1^	Yes (27%, *n* = 40)	4 (19%)	4 (3%)	**-**
	No (12%, *n* = 17)	5 (24%)	27 (22%)	
	Unknown (61%, *n* = 89)	12 (57%)	94 (75%)	

Values are presented as frequences with percentages or medians. Statistical differences were assessed using Chi-squared test ^1^ (focality, capsular invasion, vascular invasion, lymphatic invasion, tumor necrosis, desmoplasia, high risk), Mann–Whitney test ^2^ (size, number of mitoses, Ki67). “-” The *p*-value was not calculated due to insufficient numbers in some categories.

**Table 6:** Differences in disease progression between patients according to the presence of distant metastases.

Patients with MTC		Metastases 14% (*n* = 21)	No Metastases 86% (*n* = 125)	*p*
Ct at 6–12 months ^2^	pg/mL (median)	246 (*n* = 16)	26 (*n* = 115)	**<0.001**
BFS ^2^	Months (median)	0 (*n* = 21)	341 (*n* = 124)	**<0.001**
Death ^1^	Yes (16%, *n* = 23)	10 (50%)	13 (10%)	**<0.001**
	No (84%, *n* = 122)	10 (50%)	112 (90%)	
Cause of death ^1^	Cancer progression (48%, *n* = 11)	9 (90%)	2 (15%)	**0.001**
	Others (52%, *n* = 12)	1 (10%)	11 (85%)	

Values are presented as frequences with percentages or medians. Statistical differences were assessed using Chi-squared test ^1^ (Ct at 6–12 moths, BFS), Mann–Whitney test ^2^ (death, cause of death). Ct: calcitonin; BFS: biochemical-free survival.

**Table 7:** Multivariate analysis of factors associated with the development of distant metastases in patients with MTC (*n* = 131).

Variables	Odds Ratio [CI]	*p*-Valor	R Squared	−2log of Likelihood
Constant	0.018	**<0.001**	38.9	64.28
LNR	16.460 [2.879; 94.093]	**<0.001**		
Ct ≧ 500 pg/mL	7.985 [1.571; 40.594]	0.062		

Values are presented odds ratios and CI. Statistical analysis: Multivariate logistic regression. LNR: Lymph node ratio; Ct: calcitonin; CI: confidence interval.

**Table 8:** Multivariate analysis of factors associated with the development of distant metastases in patients with familiar MTC.

Variables	Odds Ratio [CI]	*p*-Valor	R Squared	−2log of Likelihood
Constant	0.014	**<0.001**	20.6	37.959
Ct ≧ 500 pg/mL	13.846 [1.544; 124.135]	**0.019**		

Values are presented odds ratios and CI. Statistical analysis: Multivariate logistic regression. CI: confidence interval. Ct: calcitonin.

## Data Availability

The original contributions presented in this study are included in this article/Supplementary Materials. Further inquiries can be directed to the corresponding author.

## Supplementary Materials

The following supporting information can be downloaded at: https://www.mdpi.com/article/10.3390/cancers17193193/s1, Table S1. Descriptive analysis. Table S2. Univariate and multivariate analysis of factors associated with the development of distant metastases in patients with MTC (*n* = 131).

## Abbreviations

The following abbreviations are used in this manuscript:

MCT	Medullary Thyroid Cancer
HP	Hyperparathyroidism
HPF	High-power field
Ct	Calcitonin
CEA	Carcinoembryonic antigen
CI	Confidence interval
TAC	Computed axial tomography
TT	Total thyroidectomy
LCB	Central bilateral lymphadenectomy
LLB	Bilateral lateral lymphadenectomy
LLU	Unilateral lateral lymphadenectomy
LNR	Lymph node ratio
BFS	Biochemical-free survival
OS	Overall survival

## References

[1] Prinzi A., Frasca F., Russo M., Le Moli R., Belfiore A., Malandrino P. (2023). Lymph Node Ratio as a Predictive Factor of Persistent/Recurrent Disease in Patients With Medullary Thyroid Cancer: A Single-Center Retrospective Study. Endocr. Pr..

[2] Siironen P., Hagström J., Mäenpää H.O., Louhimo J., Arola J., Haglund C. (2016). Lymph node metastases and elevated postoperative calcitonin: Predictors of poor survival in medullary thyroid carcinoma. Acta Oncol..

[3] Papachristos A.J., ENicholls L., Mechera R., Aniss A.M., Robinson B., Clifton-Bligh R., Gill A.J., Learoyd D., Sidhu S.B., Glover A. (2023). Management of Medullary Thyroid Cancer: Patterns of Recurrence and Outcomes of Reoperative Surgery. Oncologist.

[4] Chen Z., Mao Y., You T., Chen G. (2023). Establishment and validation of a nomogram model for predicting distant metastasis in medullary thyroid carcinoma: An analysis of the SEER database based on the AJCC 8th TNM staging system. Front. Endocrinol..

[5] Hassan A., Siddique M., Riaz S., Khan A.I., Nawaz M.K., Bashir H. (2018). Medullary Thyroid Carcinoma: Prognostic Variables And Tumour Markers Affecting Survival. J. Ayub Med. Coll. Abbottabad.

[6] Schlumberger M., Bastholt L., Dralle H., Jarzab B., Pacini F.S.J. (2012). 2012 European Thyroid Association Guidelines for Metastatic Medullary Thyroid Cancer. Endocr. Surg..

[7] Galofré J.C., Sandi J.S., Capdevila J., González E.N., Llopis C.Z., Asensio T.R.Y.C., Sáez J.M.G., Jiménez-Fonseca P., Eizaguirre G.R., Grande E. (2015). Consensus on the management of advanced medullary thyroid carcinoma on behalf of the Working Group of Thyroid Cancer of the Spanish Society of Endocrinology (SEEN) and the Spanish Task Force Group for Orphan and Infrequent Tumors (GETHI). Clin. Transl. Oncol..

[8] Machens A., Lorenz K., Weber F., Dralle H. (2021). Metastatic Risk Profile of Microscopic Lymphatic and Venous Invasion in Medullary Thyroid Cancer. Horm. Metab. Res..

[9] Chen L., Wang Y., Zhao K., Wang Y., He X. (2020). Postoperative nomogram for predicting cancer-specific and overall survival among patients with medullary thyroid cancer. Int. J. Endocrinol..

[10] Hyer S., Vini L., A’Hern R., Harmer C. (2000). Medullary thyroid cancer: Multivariate analysis of prognostic factors influencing survival. Eur. J. Surg. Oncol..

[11] Ito Y., Miyauchi A., Kihara M., Higashiiyama T., Fukushima M., Miya A. (2018). Static Prognostic Factors and Appropriate Surgical Designs for Patients with Medullary Thyroid Carcinoma: The Second Report from a Single—Institution Study in Japan. World J. Surg..

[12] Rozenblat T., Hirsch D., Robenshtok E., Grozinsky-Glasberg S., Gross D.J., Mazeh H., Benbassat C., Twito O., Levy S., Mizrachi A. (2020). The prognostic value of lymph node ratio in Medullary thyroid carcinoma: A multi-center study. Eur. J. Surg. Oncol..

[13] Lee C.R., Lee S., Son H., Ban E., Kang S.-W., Lee J., Jeong J.J., Nam K.-H., Chung W.Y., Park C.S. (2016). Medullary thyroid carcinoma: A 30-year experience at one institution in Korea. Ann. Surg. Treat. Res..

[14] Kotwal A., Erickson D., Geske J.R., Hay I.D., Castro M.R. (2021). Predicting Outcomes in Sporadic and Hereditary Medullary Thyroid Carcinoma over Two Decades. Thyroid.

[15] Mathiesen J.S., Kroustrup J.P., Vestergaard P., Stochholm K., Poulsen P.L., Rasmussen Å.K., Feldt-Rasmussen U., Schytte S., Londero S.C., Pedersen H.B. (2019). Survival and Long-Term Biochemical Cure in Medullary Thyroid Carcinoma in Denmark 1997–2014: A Nationwide Study. Thyroid.

[16] Qu N., Shi R.-L., Luo T.-X., Wang Y.-L., Li D.-S., Wang Y., Huang C.-P., Ji Q.-H. (2016). Prognostic significance and optimal cutoff of age in medullary thyroid cancer. Oncotarget.

[17] Sahli Z.T., Canner J.K., Zeiger M.A., Mathur A. (2021). Association between age and disease specific mortality in medullary thyroid cancer. Am. J. Surg..

[18] Tang J., Jiang S., Gao L., Xi X., Zhao R., Lai X., Zhang B., Jiang Y. (2021). Construction and Validation of a Nomogram Based on the Log Odds of Positive Lymph Nodes to Predict the Prognosis of Medullary Thyroid Carcinoma After Surgery. Ann. Surg. Oncol..

[19] Esfandiari N.H., Hughes D.T., Yin H., Banerjee M., Haymart M.R. (2014). The effect of extent of surgery and number of lymph node metastases on overall survival in patients with medullary thyroid cancer. J. Clin. Endocrinol. Metab..

[20] Kandil E., Gilson M.M., Alabbas H.H., Tufaro A.P., Dackiw A., Tufano R.P. (2011). Survival implications of cervical lymphadenectomy in patients with medullary thyroid cancer. Ann. Surg. Oncol..

[21] Prassas D., Kounnamas A., Cupisti K., Schott M., Knoefel W.T., Krieg A. (2022). Prognostic Performance of Alternative Lymph Node Classification Systems for Patients with Medullary Thyroid Cancer: A Single Center Cohort Study. Ann. Surg. Oncol..

[22] Shao Y., Li G., Wei T., Gong R., Li Z., Zhu J., Lei J. (2022). Distant metastasis in medullary thyroid carcinoma: Clinical outcomes and implications of T stage. Clin. Endocrinol..

[23] Gülben K., Berberoğlu U., Boyabatlı M. (2006). Prognostic factors for sporadic medullary thyroid carcinoma. World J. Surg..

[24] Le M.-K., Kawai M., Odate T., Vuong H.G., Oishi N., Kondo T. (2022). Metastatic Risk Stratification of 2526 Medullary Thyroid Carcinoma Patients: A Study Based on Surveillance, Epidemiology, and End Results Database. Endocr. Pathol..

[25] Pazaitou-Panayiotou K., Chrisoulidou A., Mandanas S., Tziomalos K., Doumala E., Patakiouta F. (2014). Predictive factors that influence the course of medullary thyroid carcinoma. Int. J. Clin. Oncol..

[26] Su H., Men Q., Hao J., Zhang F. (2024). Risk factor analysis of distant metastases in patients with primary medullary thyroid cancer: A population-based study. Eur. Arch. Oto-Rhino-Laryngol..

[27] Xu B., Fuchs T.L., Ahmadi S., Alghamdi M., Alzumaili B., Bani M.-A., Baudin E., Chou A., De Leo A., Fagin J.A. (2022). International Medullary Thyroid Carcinoma Grading System: A Validated Grading System for Medullary Thyroid Carcinoma. J. Clin. Oncol..

[28] Wells S.A., Asa S.L., Dralle H., Elisei R., Evans D.B., Gagel R.F., Lee N., Machens A., Moley J.F., Pacini F. (2015). Revised American thyroid association guidelines for the management of medullary thyroid carcinoma. Thyroid.

[29] Schiettecatte J., Strasser O., Anckaert E., Smitz J. (2016). Performance evaluation of an automated electrochemiluminescent calcitonin (CT) immunoassay in diagnosis of medullary thyroid carcinoma. Clin. Biochem..

[30] Febrero B., Rodríguez J.M., Ríos A., Segura P., Pérez-Sánchez B., Torregrosa N., Hernández A.M., Parrilla P. (2019). Prophylactic thyroidectomy in multiple endocrine neoplasia 2 (MEN2) patients with the C634Y mutation: A long-term follow-up in a large single-center cohort. Eur. J. Surg. Oncol..

[31] Lifante J., Blanchard C., Mirallié E., David A., Peix J. (2014). Role of preoperative basal calcitonin levels in the timing of prophylactic thyroidectomy in patients with germline RET mutations. World J. Surg..

[32] Baloch Z.W., Asa S.L., Barletta J.A., Ghossein R.A., Juhlin C.C., Jung C.K., LiVolsi V.A., Papotti M.G., Sobrinho-Simões M., Tallini G. (2022). Overview of the 2022 WHO Classification of Thyroid Neoplasms. Endocr. Pathol..

[33] Koperek O., Scheuba C., Cherenko M., Neuhold N., De Micco C., Schmid K.W., Niederle B., Kaserer K. (2008). Desmoplasia in medullary thyroid carcinoma: A reliable indicator of metastatic potential. Histopathology.

[34] Brierley J., Gospodarowicz M.K., Witterkind C.H.E. (2017). TNM Classification of Malignant Tumors.

[35] Machens A., Dralle H. (2010). Biomarker-based risk stratification for previously untreated medullary thyroid cancer. J. Clin. Endocrinol. Metab..

[36] Kim J., Park J., Park H., Choi M.S., Jang H.W., Kim T.H., Kim S.W., Chung J.H. (2021). Metastatic lymph node ratio for predicting recurrence in medullary thyroid cancer. Cancers.

[37] Hao W., Zhao J., Guo F., Gu P., Zhang J., Huang D., Ruan X., Zeng Y., Zheng X., Gao M. (2023). Value of lymph node ratio as a prognostic factor of recurrence in medullary thyroid cancer. PeerJ.

[38] Zheng G., Liu J., Xu H., Dong C., Cao X., He Q., Zhang G., Wang W., Wang L., Yang X. (2025). Prognostic factors for progression free survival in patients with medullary thyroid cancer: A multicenter cohort study. Updat. Surg..

[39] Jiang T., Huang C., Xu Y., Su Y., Zhang G., Xie L., Huang L., You S., Zha J. (2017). Ratio of positive lymph nodes: The prognostic value in stage IV thyroid cancer. Oncotarget.

[40] Niederle M.B., Riss P., Selberherr A., Koperek O., Kaserer K., Niederle B., Scheuba C. (2021). Omission of lateral lymph node dissection in medullary thyroid cancer without a desmoplastic stromal reaction. Br. J. Surg..

[41] Machens A., Kaatzsch P., Lorenz K., Horn L.-C., Wickenhauser C., Schmid K.W., Dralle H., Siebolts U. (2022). Abandoning node dissection for desmoplasia-negative encapsulated unifocal sporadic medullary thyroid cancer. Surgery.

