# Supplementary material for: Predictors of Distant Metastasis in Patients with Medullary Thyroid Carcinoma
Source: Cancers (Basel). 2025 Sep 30;17(19):3193. doi: 10.3390/cancers17193193 (PMC12524247; doi:10.3390/cancers17193193)
Supplement: Supplementary file 1 [file cancers-17-03193-s001.zip › cancers-3860909-supplementary.pdf]

cancers MDPI

Suplemmentary tables

Table S1. Descriptive analysis.

Sex	Woman (51%, n=75)<br>Man (49%, n=71)
Age at diagnosis	Ages (Median)
Heredability	Sporadic (25%, n=37)<br>Familial (75%, n=109)
Ct	242 pg/mL, 52-1000 (median, RIQ)
CEA	7.4ng/mL, 1.95-29 (median, RIQ)
Thyroid ultrasound	Unilateral nodule/s (42%, n=52)<br>Bilateral nodules/s (30%, n=37)<br>Normal (28%, n=35)<br>Suspicious adenopathy (19%, n=28)<br>No (66%, n=96)<br>Unknown (15%, n=22)
Size in the ultrasound	15 Millimeters, 9.5- 20.5 (median, RIQ)
Phenotype- Feocromocitoma/ paraganglioma	Yes (58%, n=63)<br>No (42%, n=46)
Phenotype HP	Yes (12%, n=13)<br>No (88%, n=96)
Genetic mutation	Very high risk (3%, n=3)<br>High risk (92%, n=98)<br>Moderate risk (5%, n=6)
Type of genetic mutation	Exon 11, pC634T (75%, n=80)<br>Exon 11, pC634Y (9%, n=10)<br>Exon 11, pC634A (5%, n=6))<br>Exon 14; pV804M (3%, n=3)<br>Exon 16; pM918T (3%, n=3)<br>Exon 11, pC634R (2%, n=2)<br>Exon 10, pC618A (1%, n=1)<br>Exon10; pC618P (1%, n=1)<br>Exon 13; pL790F (1%, n=1)
Treatment	Early/ prophylactic (25%, n=36)<br>Curative (72%, n=105)<br>Palliative (3%, n=5)

Surgical technique	TT (33%, n=48)<br>TT + CLND (22%, n=33)<br>TT + CLND + ULLND (8%, n=12)<br>TT+ CLND + BLLND (36%, n=52)<br>Somatostatin analogues (1%, n=1)
Complications	Yes (33%, n=48)<br>No (67%, n=98)
Hypoparathyroidism	Transitory (21%, n=30)<br>Permanent (8%, n=13)<br>No (71%, n=103)
Recurrent laryngeal nerve paralysis	Transitory (3.5%, n=5)<br>Permanent (3.5%, n=5)<br>No (93%, n=134)
T	T1-2 (88%, n=128)<br>T3-4 (12%, n=17)
N	N0 (28%, n=41)<br>N1a (9%, n=13)<br>N1b (26%, n=38)<br>Nx (37%, n=54)
Nodal involvement	Yes (35%, n=51)<br>No/no lymphadenectomy (65%, n=95)
Central nodal involvement	Yes (15%, n=15)<br>No (85%, n=82)
Ipsilateral lateral nodal involvement	Yes (36%, n=35)<br>No (64%, n=63)
Contralateral lateral nodal involvement	Yes (35%, n=18)<br>No (65%, n=34)
Stage	Stage I y II (63%, n=92)<br>Stage III y IV (37%, n=54)
Number of lymph nodes removed	17, 5-31 (median, RIQ)
Number of pathological lymph nodes removed	1, 0-4 (median, RIQ)
LNR	0.05, 0-0.092 (median, RIQ)
Size	10 Millimeters, 5-20 (median, RIQ)
Focality	Unifocal (31%, n=46)<br>Multifocal (66%, n=95)<br>Unknown (3%, n=5)
Capsular invasion	Yes (8%, n=12)<br>No (7%, n=10)

	Unknown (85%, n=124)
Vascular invasion	Yes (13%, n=19)<br>No (32%, n=47)<br>Unknown (55%, n=80)
Lymphatic invasion	Yes (16%, n=24)<br>No (28%, n=41)<br>Unknown (56%, n=81)
Tumoral necrosis	Yes (1%, n=1)<br>No (31%, n=46)<br>Unknown (68%, n=99)
Number of mitoses	0-4 (97%, n=76)<br>≧5 (3%, n=2)
Ki-67	0-4 (88%, n=36)<br>≧5 (12%, n=5)
Desmoplasia	Yes (24%, n=35)<br>No (6%, n=9)<br>Unknown (70%, n=102)
High risk	Yes (27%, n=40)<br>No (12%, n=17)<br>Unknown (61%, n=89)
Ct at 6-12 months	29.50pg/mL, 8.8-98.5 (median, IQR)
BFS	132 months, 0-272.5 (median, IQR)
Death	Yes (16%, n=23)<br>No (84%, n=122)
Cause of the death	Tumor progression (48%, n=11)<br>Others (52%, n=12)

IQR: interquartile range; BFS: biochemical free survival, TT: total thyroidectomy. CLND: central lymph node dissection, ULLND: unilateral neck lateral dissection, BLLND: bilateral neck lateral dissection.

Table S2. Univariate and multivariate analysis of factors associated with the development of distant metastases in patients with MTC (n=131).

	**Univariate analysis**		**Multivariate analysis**	
**Variables**	**Odds Ratio [CI]**	**p-valor**	**Odds Ratio [CI]**	**p-valor**
Age at diagnosis	1.035 [1.007; 1.063]	0.013		
Heredity	4.889 [1.828-13.078]	0.002		
Ct ≧500pg/mL	17.292 [3.758-79.567]	<0.001	7.985 [1.571; 40.594]	**0.062**
Tumor size	1.045 [1.018-1.071]	0.001		
T	6.708 [2.158-20.854]	0.001		
N	1.995 [1.307-3.045]	0.001		
Lymph node involvement	8.324 [1.776-39.014]	0.007		
Ipsilateral lateral lymph node involvement	8.155 [2.928-22.715]	<0.001		
Contralateral lymph node involvement	5.531 [1.827-16.750]	0.002	16.460 [2.879; 94.093]	**<0.001**
LNR	34.338 [7.233-163.008]	<0.001		
Stage	9.158 [2.871-29.214]	<0.001		

Values are presented odds ratios and CI. Statistical analysis: Multivariate logistic regression. CI: 1confidence interval.
